# Advanced cardiac magnetic resonance imaging for assessment of obstructive coronary artery disease—ADVOCATE-CMR study rationale and design

**DOI:** 10.1016/j.jocmr.2025.101900

**Published:** 2025-04-25

**Authors:** Sonia Borodzicz-Jazdzyk, Geoffrey W. de Mooij, Alexander W. den Hartog, Mark B.M. Hofman, Marco J.W. Götte

**Affiliations:** a Department of Cardiology, Amsterdam UMC, Vrije Universiteit Amsterdam, Amsterdam Cardiovascular Sciences, de Boelelaan 1117, 1081 HV Amsterdam, the Netherlands; b 1st Department of Cardiology, Medical University of Warsaw, Banacha 1a, 02-097 Warsaw, Poland; c Department of Radiology and Nuclear Medicine, Amsterdam UMC, Vrije Universiteit Amsterdam, de Boelelaan 1118, 1081 HV, Amsterdam, the Netherlands

**Keywords:** First-pass perfusion cardiovascular magnetic resonance, Quantitative perfusion, Stress T1 mapping reactivity, Oxygenation-sensitive cardiovascular magnetic resonance, Coronary artery disease

## Abstract

**Background:**

First-pass stress-perfusion cardiovascular magnetic resonance (CMR) imaging is the guidelines-recommended non-invasive test for the detection of obstructive coronary artery disease (CAD). Recently developed quantitative perfusion CMR (QP CMR) allows quantification of myocardial blood flow. Moreover, the latest developments established several methods of CAD assessment without the need for a contrast agent, including stress T1 mapping reactivity (∆T1) and oxygenation-sensitive CMR (OS-CMR). These methods might eliminate the need for contrast administration in clinical practice, reducing time, invasiveness, and costs, thereby simplifying the evaluation of patients with suspected obstructive CAD. The ADVOCATE-CMR study aims to validate QP CMR, ∆T1, and OS-CMR imaging against invasive fractional flow reserve (FFR) for the detection of obstructive CAD. The study also aims to head-to-head compare the diagnostic accuracy of these CMR techniques with the conventional visual assessment of stress-perfusion CMR and to correlate them to short- and long-term clinical outcomes.

**Study design and Methodology:**

ADVOCATE-CMR is a single-center, observational, prospective, cross-sectional cohort study. The study will enroll 182 symptomatic patients with suspected obstructive CAD scheduled for invasive coronary angiography (ICA). Before ICA, all participants will undergo CMR imaging, including OS-CMR with breathing maneuvers, rest, and adenosine stress T1 mapping and rest and adenosine stress first-pass perfusion. Subsequently, ICA will be performed, including FFR, instantaneous wave-free ratio, resting Pd/Pa, coronary flow reserve, and index of microvascular resistance measurements in all main coronary arteries. A follow-up CMR scan with the same protocol will be performed at 3 months after ICA. Clinical follow-up will be performed at 3, 6 months, 1 and 3 years after ICA.

**Conclusion:**

The ADVOCATE-CMR will be the first study comprehensively evaluating and comparing head-to-head the diagnostic performance of a range of contrast- and non-contrast agent-based CMR imaging methods (including QP CMR, ∆T1, and OS-CMR) for the detection of FFR-defined obstructive CAD. We expect to establish a validated and time-efficient diagnostic workflow available to a wide range of general CMR services. Finally, these improvements may enable CMR to become an effective non-invasive, radiation-free gatekeeper for ICA in patients with suspected obstructive CAD, potentially without the need for a contrast agent.

## Graphical abstract

**Figure:**
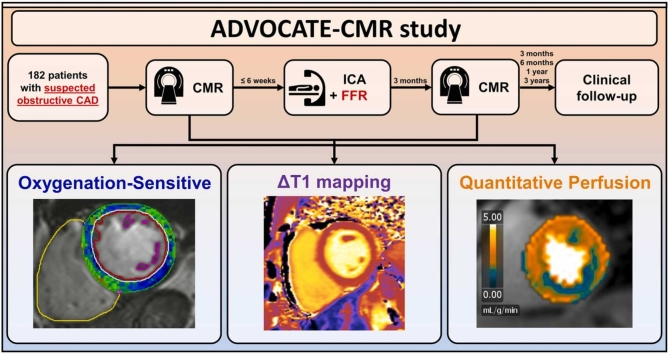

## 1 Background

Obstructive coronary artery disease (CAD) involves the formation of atherosclerotic plaques in the lumen of coronary arteries, which causes a demand-supply mismatch of oxygen. Consequently, this leads to myocardial ischemia, reflected by recurrent, transient episodes of chest pain (angina pectoris) [1]. Ischemic heart disease affects around 126 million people worldwide and is the leading global cause of mortality [2], [3], [4]. Early diagnosis of obstructive CAD enables fast initiation of targeted treatment, improves patient quality of life, facilitates rehabilitation, accelerates fast return to the previous socio-economic environment, and reduces costs for society [5], [6].

Invasive coronary angiography (ICA) is regarded as the “gold standard” to detect obstructive CAD, and multiple trials have demonstrated that coronary revascularizations should be ischemia-driven, as measured by fractional flow reserve (FFR), to relieve symptoms and improve outcome [7], [8], [9], [10]. However, despite symptoms, in almost two-thirds of women and one-third of men, ICA does not show obstructive CAD [11], [12], [13]. Therefore, up to 60% of the current diagnostic ICA could be avoided, preventing patients from getting exposed to potential ICA-related risks. Therefore, a reliable non-invasive screening tool to discriminate patients with obstructive CAD from patients without obstructive CAD will prevent unnecessary ICA, thereby reducing costs and increasing efficient use of catheterization laboratories for more targeted procedures. Among others, first-pass stress-perfusion cardiovascular magnetic resonance (CMR) has been proposed as the non-invasive method of choice for the detection of obstructive CAD due to its high spatial resolution, accurate assessment of myocardial viability and function, cost-effectiveness, and lack of ionizing radiation [6], [14], [15], [16], [17], [18], [19]. In clinical routine, first-pass stress-perfusion CMR images are visually analyzed. This approach is based on the evaluation of the arrival and first passage of a gadolinium-based contrast agent (GBCA) through the left ventricular myocardium and the identification of regions with relatively lower signal intensity (SI), indicating hypoperfusion, as a surrogate of myocardial ischemia [20]. However, visual assessment of stress-perfusion CMR may underestimate the extent of perfusion defect in patients with multivessel disease and has poor diagnostic accuracy for the detection of coronary microvascular dysfunction (CMD) [21], [22].

Recently, conventional visual assessment of first-pass perfusion CMR has been substantially improved by quantification of myocardial blood flow (MBF) and the creation of color pixel perfusion maps (quantitative perfusion CMR [QP CMR]) [23], [24], [25], [26], [27], [28], [29], [30], [31]. MBF is quantified by tracer kinetic analysis of the SI curves of GBCA first pass. Until recently, QP CMR required cumbersome and time-consuming manual processing to delineate myocardial regions of interest (ROIs), which made it prone to interobserver variability and prevented its widespread use in clinical routine. To address these limitations, a fully automated, pixel-wise QP CMR image processing framework has been established, which is reproducible, time-efficient, and does not require manual delineation of ROIs and myocardial image segmentation [26]. Fully automated pixel-wise QP CMR has also shown high diagnostic performance for detecting ICA- and/or FFR-confirmed significant CAD [25], [26], [30], [32], [33]. However, QP CMR is still predominantly a research tool due to substantial inter-study differences in acquisition and analysis methods and the lack of established reference standards [34], [35], [36], [37]. Therefore, further research is needed to propose algorithms ready to be used in daily clinical routine in patients with suspected obstructive CAD [32], [38].

However, it must be noted that GBCA-based QP CMR, despite its advantages over the conventional visual assessment approach, is associated with some clinically relevant drawbacks. First-pass perfusion CMR imaging (either conventional or QP) requires administration of GBCA, which for this purpose in most countries is used off-label. In addition, GBCAs are advised not to be used in patients with severe renal dysfunction and have been associated with the rare development of nephrogenic systemic fibrosis. Furthermore, its repeated administration may cause accumulation of gadolinium in brain tissues, albeit of unknown clinical significance [39]. Therefore, there are still some regulations and safety concerns regarding the use of GBCA [40]. Due to these matters, the development and clinical validation of alternative, accurate methods for CMR ischemia detection without the need for GBCA are warranted. Recently, novel, non-GBCA-based CMR imaging strategies have been proposed for evaluation of myocardial ischemia, including stress T1 mapping reactivity (∆T1) and oxygenation-sensitive CMR (OS-CMR). These methods, however, have not been introduced into diagnostics and clinical decision-making in patients with CAD due to the lack of proper validation against the invasive gold standard measures in a real-world population.

**Fig. 1:**
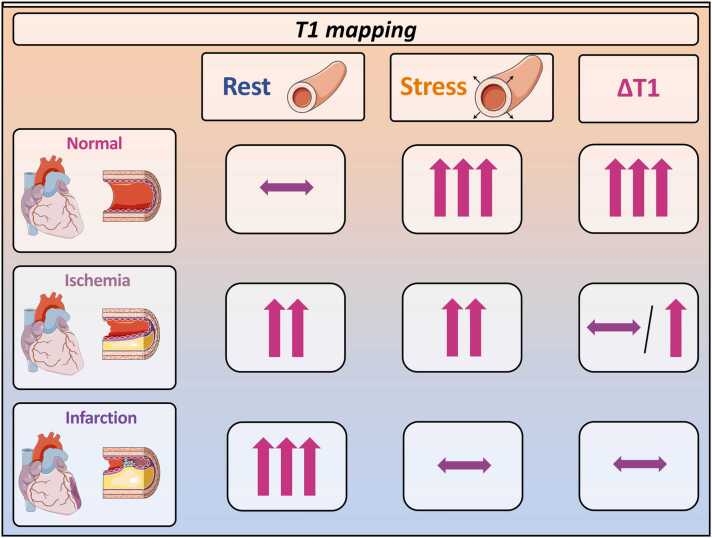
Native T1 mapping and T1 mapping reactivity in normal myocardium, myocardial ischemia, and infarction in rest and vasodilation-induced stress. *ΔT1* stress T1 mapping reactivity

Native T1 mapping is a non-contrast agent technique that quantifies the longitudinal relaxation time constant (T1). T1 prolongs in the heart as a result of an increased interstitial space but also because of an increased myocardial free water content. Therefore, T1 mapping may reflect the total myocardial blood volume (MBV) localized in the arteries, capillaries, veins, and myocardium [41], [42], [43], [44]. Obstructive CAD is associated with changes in MBV and therefore, T1 mapping technique has been proposed as a contrast agent-free method to evaluate the presence of hemodynamically significant coronary stenosis. From a pathophysiological point of view, coronary artery stenosis increases vascular resistance, which in intermediate lesions can be compensated by microvascular dilation [45]. In this autoregulatory mechanism, capillaries vasodilate counterbalancing the stenosis severity, which preserves sufficient myocardial perfusion. This vasodilation results in increased stationary MBV and resting native T1 mapping values within the myocardium [44], [45], [46]. Exogenous administration of a vasodilator during stress imaging studies may further dilate microvasculature, and increase MBV and native T1 mapping values. However, in the presence of coronary stenosis, where resting MBV and native T1 values are already high, the difference between rest and stress MBV, thus difference in T1 values (∆T1), will be lower than in normal physiology (Fig. 1). Therefore, measurement of differences in T1 values between vasodilator-induced stress and at rest (∆T1) may identify the degree of MBV increase, which reflects microvascular reserve and the true physiological impact of the stenosis on the downstream coronary circulation [44], [47], [48], [49]. Indeed, several clinical studies have confirmed that ischemic myocardium has higher resting T1 values and blunted ∆T1 (i.e., low difference between already high rest and vasodilator-induced slightly increased T1 values), which is not observed in remote, non-ischemic myocardium (i.e., substantial increase in vasodilator-induced T1 values). Moreover, infarcted myocardial tissue shows even higher resting T1 values in comparison to ischemic myocardium, in the absence of T1 reactivity [47], [49], [50], [51]. Although ∆T1 has been suggested to be a promising non-invasive, non-GBCA-dependent biomarker of myocardial ischemia, its diagnostic accuracy has not been validated against gold standard invasive measures, which prevents its wide clinical adoption.

**Fig. 2:**
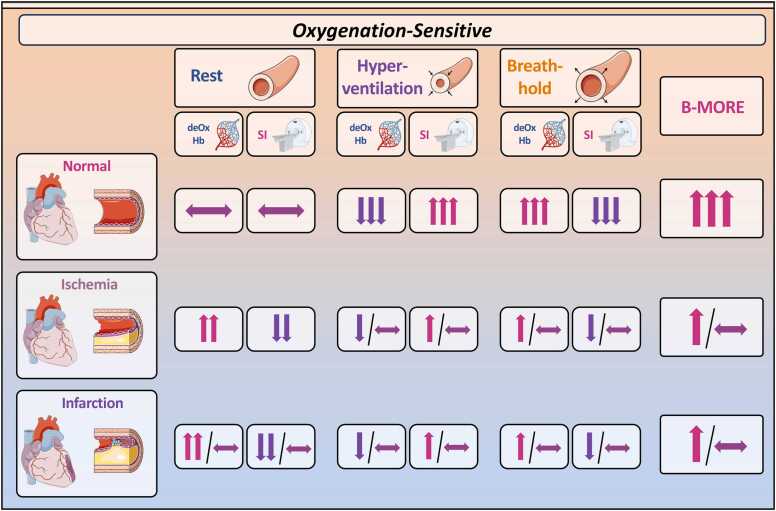
Myocardial oxygenation-sensitive signal intensity in normal, ischemic, and infarcted myocardium in rest and stress-induced vasodilation. *B-MORE* breathing-induced myocardial oxygenation reserve, *deOX-Hb* deoxygenated hemoglobin, *SI* signal intensity

OS-CMR is a technique that non-invasively evaluates myocardial oxygenation without the need for a GBCA. When performed with breathing maneuvers, OS-CMR may become a needle-free, safe, and cost-effective CMR approach for detection of myocardial ischemia. OS-CMR uses the blood-oxygen-level-dependent (BOLD) phenomenon where the deoxygenated hemoglobin acts as an intrinsic paramagnetic contrast agent, enhancing spin-spin interaction, accelerating the transverse magnetization decay, and shortening the spin-spin relaxation times (T2 and T2*) [52], [53]. Therefore, increased levels of deoxygenated hemoglobin within the myocardium lead to shortening of T2* relaxation time, which can be visualized through lower SI in T2*-weighted images or T2* maps [54]. Since myocardial ischemia is directly related to increased levels of deoxygenated hemoglobin in the capillaries, it can be detected as a reduction in SI on OS images. Conversely, an increase in the levels of oxygenated hemoglobin (i.e., decreased amount of deoxygenated hemoglobin) increases SI in the T2*-weighted images (Fig. 2) [54], [55], [56], [57]. Indeed, several relatively small clinical studies have demonstrated that OS-CMR has the potential to effectively detect obstructive CAD [58], [59], [60], [61], [62].

For a needle-free diagnostic test for assessing coronary artery function, including the presence of obstructive CAD, OS-CMR has been combined with vasoactive breathing maneuvers, i.e., hyperventilation and breath-hold. These maneuvers lead to differences in blood concentration of CO_2_, which is a potent physiological vasodilator [63]. Accordingly, hyperventilation mildly increases levels of oxygenated hemoglobin but especially decreases capillary CO_2_, which induces vasoconstriction and decreases myocardial SI in OS images. To the contrary, in the absence of CAD, a long breath-hold increases capillary CO_2_, inducing vasodilation, and increasing myocardial SI accordingly (Fig. 2) [64]. Therefore, measurement of the breathing-induced myocardial oxygenation reserve (B-MORE), defined as the relative increase in myocardial oxygenation during a post-hyperventilation breath-hold, or other markers of changes in myocardial oxygenation as observed in OS-CMR images, may serve as an accurate non-invasive biomarker of downstream vascular function. Although OS-CMR with breathing maneuvers as a non-invasive, non-GBCA-based and therefore needle-free method for the detection of coronary vascular dysfunction is promising, it still needs to be properly validated against invasive gold standard measures and other non-invasive imaging modalities before it can be introduced on a wide scale into clinical routine.

The primary objective of ADVOCATE-CMR study is to validate the new, non-invasive QP CMR imaging against invasive gold standard FFR for the detection of obstructive CAD. The main secondary and tertiary objectives are to validate ∆T1 and OS-CMR against FFR for detection of obstructive CAD, to compare (head-to-head) the diagnostic accuracy of the QP CMR, ∆T1, and OS-CMR imaging with the conventional visual assessment of GBCA-based first-pass perfusion imaging to detect obstructive CAD, to correlate these new CMR parameters to short- and long-term clinical outcomes and to study the change in myocardial perfusion, MBV, and myocardial oxygenation after coronary revascularization.

## 2 Methods

### 2.1 Study design

ADVOCATE-CMR is a single-center, observational, prospective, cross-sectional cohort study performed at the Amsterdam University Medical Centers—Location VUmc. The study is performed in accordance with the principles of the Declaration of Helsinki, and all patients need to provide informed written consent to participate. The study protocol has been approved by the local ethics committee (Medisch Ethische Toetsingscommissie Amsterdam UMC) and is registered in ClinicalTrials.gov under the number NCT06419894.

**Fig. 3:**
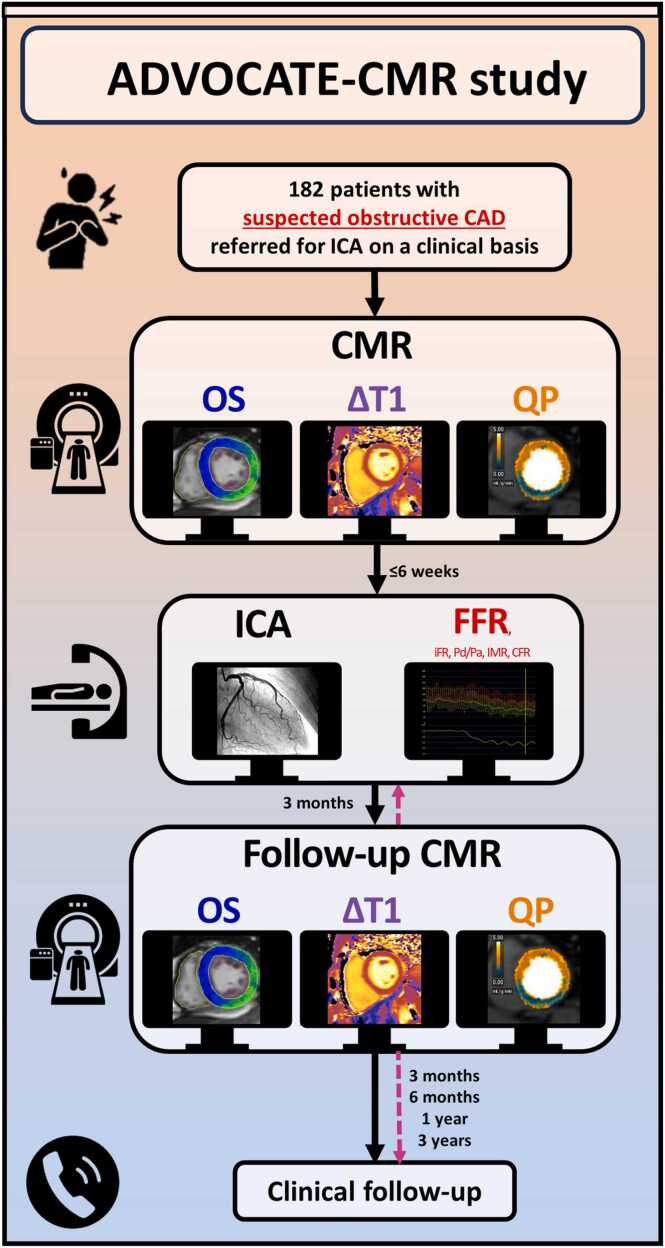
ADVOCATE-CMR study flowchart. *ΔT1* stress T1 mapping reactivity, *CAD* coronary artery disease, *CFR* coronary flow reserve, *CMR* cardiac magnetic resonance, *FFR* fractional flow reserve, *ICA* invasive coronary angiography, *iFR* instantaneous wave-free ratio, *IMR* index of microvascular resistance, *OS* oxygenation-sensitive, *Pd/Pa* ratio between proximal and distal coronary pressures over entire resting cycle period, *QP* quantitative perfusion

The flowchart of the study is presented in Fig. 3. The study will enroll 182 symptomatic patients with suspected obstructive CAD scheduled for ICA according to the decision of the treating clinician (see Section 2.9). Before ICA, all study participants will undergo CMR imaging, including rest and adenosine stress perfusion, rest and adenosine stress T1 mapping, and OS-CMR with breathing maneuvers. Subsequently, within 6 weeks, ICA will be performed including FFR, instantaneous wave-free ratio (iFR), resting ratio between proximal and distal coronary pressures over the entire resting cycle period (Pd/Pa), coronary flow reserve (CFR), and index of microvascular resistance (IMR) measurements in all main coronary arteries. A second follow-up CMR scan according to the same scan protocol as the first CMR scan will be performed at 3 months after ICA (or 3 months after revascularization, if performed separately more than 1 day following ICA). Clinical follow-up will be at 3, 6 months, 1 and 3 years after the ICA (or revascularization if applicable).

### 2.2 Study endpoints

Primary, secondary, and tertiary endpoints are presented in Table 1.

**Table 1:** ADVOCATE-CMR study endpoints.

*Primary endpoint*
- Diagnostic accuracy (sensitivity, specificity, accuracy, AUC, PPV, NPV) of QP CMR (stress MBF, stress rMBF, MPR, and rMPR) to detect obstructive CAD, as defined by FFR
*Secondary endpoints*
- Diagnostic accuracy (sensitivity, specificity, accuracy, AUC, PPV, NPV) of ΔT1 to detect obstructive CAD, as defined by FFR - Diagnostic accuracy (sensitivity, specificity, accuracy, AUC, PPV, NPV) of OS-CMR (B-MORE) to detect obstructive CAD, as defined by FFR - Head-to-head comparison of diagnostic accuracies (sensitivity, specificity, accuracy, AUC, PPV, NPV) of QP CMR (stress MBF, stress rMBF, MPR, rMPR), ΔT1, OS-CMR, and conventional visual assessment of GBCA-based first-pass perfusion imaging to detect obstructive CAD, as defined by FFR - Diagnostic accuracy (sensitivity, specificity, accuracy, AUC, PPV, NPV) of QP CMR (stress MBF, stress rMBF, MPR, and rMPR) to detect obstructive CAD, as defined by iFR and resting Pd/Pa - Diagnostic accuracy (sensitivity, specificity, accuracy, AUC, PPV, NPV) of ΔT1 to detect obstructive CAD, as defined by iFR and resting Pd/Pa - Diagnostic accuracy (sensitivity, specificity, accuracy, AUC, PPV, NPV) of OS-CMR (B-MORE) to detect obstructive CAD, as defined by iFR and resting Pd/Pa - Head-to-head comparison of diagnostic accuracies (sensitivity, specificity, accuracy, AUC, PPV, NPV) of QP CMR (stress MBF, stress rMBF, MPR, rMPR), ΔT1, OS-CMR, and conventional visual assessment of GBCA-based first-pass perfusion imaging to detect obstructive CAD, as defined by iFR and resting Pd/Pa - Relation of stress and rest MBF and rMBF, MPR and rMPR, ΔT1 and OS-CMR markers to SAQ-7 Summary score, SAQ-7 Angina Frequency score, SAQ-7 Physical Limitation score, SAQ-7 Quality of Life score (before and after revascularization), and Rose Dyspnea Scale score - Prognostic value of QP CMR (stress MBF, stress rMBF, MPR, and rMPR), stress T1 mapping reactivity and OS-CMR in predicting occurrence of a 1) composite of cardiovascular death, myocardial infarction, ischemia-driven coronary revascularization or stroke; 2) composite of myocardial infarction or ischemia-driven coronary revascularization; 3) composite of cardiovascular death, stroke or myocardial infarction; 4) myocardial infarction; 5) ischemia-driven coronary revascularization; 6) stroke; 7) death from any cause; 8) cardiovascular death
*Tertiary endpoints*
- Diagnostic accuracies (sensitivity, specificity, accuracy, AUC, PPV, NPV) of QP CMR (stress MBF, stress rMBF, MPR, and rMPR), ΔT1, and B-MORE to detect CMD, as defined by CFR - Diagnostic accuracy (sensitivity, specificity, accuracy, AUC, PPV, NPV) of QP CMR (stress MBF, stress rMBF, MPR, and rMPR), ΔT1, and B-MORE to differentiate between CMD (as defined by CFR) and 3-vessel obstructive CAD - Change in stress and rest MBF and rMBF, MPR and rMPR, ΔT1 and B-MORE before and after revascularization - Costs and procedural time of QP CMR, stress T1 mapping reactivity, and OS-CMR compared to ICA

*AUC* area under the curve, *B-MORE* breathing-induced myocardial oxygenation reserve, *CAD* coronary artery disease, *CMD* coronary microvascular dysfunction, *CMR* cardiac magnetic resonance, *FFR* fractional flow reserve, *iFR* instantaneous wave-free ratio, *MBF* myocardial blood flow, *MPR* myocardial perfusion reserve, *NPV* negative predictive value, *OS-CMR* oxygenation-sensitive cardiac magnetic resonance, *Pd/Pa* ratio between proximal and distal coronary pressures over entire resting cycle period, *PPV* positive predictive value, *QP CMR* quantitative perfusion cardiac magnetic resonance, *rMBF* relative myocardial blood flow, *rMPR* relative myocardial perfusion reserve, *SAQ* Seattle Angina Questionnaire, *ΔT1* stress T1 mapping reactivity

### 2.3 Recruitment and data collection

Inclusion and exclusion criteria are presented in Table 2. Potential study subjects are recruited (from different hospitals or outpatient clinics) after they are scheduled to undergo ICA at Amsterdam UMC on a clinical basis. A registration log containing anonymized records of all screened patients, who are not enrolled due to either ineligibility or their refusal to participate, will be maintained. After enrollment, information on baseline characteristics and cardiovascular risk factors, including angina-related symptoms, function, and quality of life (assessed according to the Seattle Angina Questionnaire-7 [SAQ-7]), history of present and past diseases, family history of cardiovascular and other diseases, cardiovascular risk factors, current pharmacotherapy, available results of laboratory blood tests, and other diagnostic tests will be collected.

**Table 2:** ADVOCATE-CMR inclusion and exclusion criteria.

Inclusion criteria	- Suspected obstructive CAD - No documented prior history of CAD - Clinical referral for ICA according to the referring clinician’s decision - Competent adult (age ≥18 years) - Signed informed consent
Exclusion criteria	- Acute coronary syndrome - History of coronary revascularization (percutaneous coronary intervention or coronary artery bypass grafting surgery) - History of CAD or acute coronary syndrome (myocardial infarction, unstable angina) - Use of sildenafil or dipyridamole that cannot be terminated - Pregnancy or lactation - Allergic reaction to iodized contrast - Concurrent or prior (within last 30 days) participation in other research studies using interventional drugs - Extensive comorbidities (i.e., cancer, other severe chronic diseases) - Contraindication for CMR with GBCA (including severe claustrophobia, MR unsafe implants/devices or MR conditional devices not suitable for 3T scanner, acute or severe renal failure with eGFR <30 mL/min/1.73 m2, known hypersensitivity for GBCA) - Contraindications for adenosine usage (including hypersensitivity to adenosine/dipyridamole/regadenoson, second- or third-degree atrio-ventricular block, sick sinus syndrome, sinus bradycardia [heart rate <40 bpm], long QT syndrome, severe hypertension [> 220/120 mmHg], systolic blood pressure <90 mmHg, concomitant use of dipyridamole, severe asthma, or severe chronic obstructive pulmonary disease)

*CAD* coronary artery disease, *CMR* cardiac magnetic resonance, *eGFR* estimated glomerular filtration rate, *GBCA* gadolinium-based contrast agent, *ICA* invasive coronary artery, *MR* magnetic resonance

### 2.4 CMR imaging

**Fig. 4:**
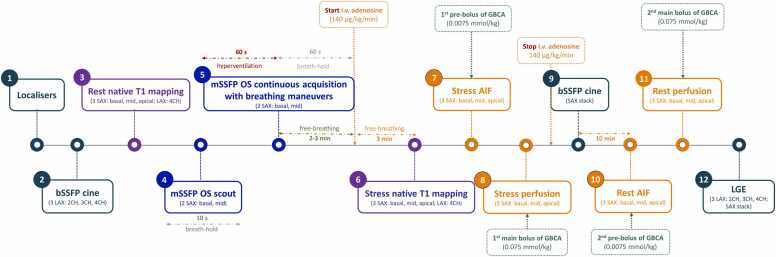
ADVOCATE-CMR imaging protocol. *2CH* two-chamber view, *3CH* three-chamber view, *4CH* four-chamber view, *AIF* arterial input function, *bSSFP* balanced steady-state free precession, *GBCA* gadolinium-based contrast agent, *i.v.* intravenously, *LAX* long axis, *LGE* late gadolinium enhancement, *mSSFP* modified steady-state free precession, *OS* oxygenation-sensitive, *SAX* short axis

All subjects included in the study will undergo adenosine stress CMR using a 3T whole-body magnetic resonance imaging (MRI) scanner (Magnetom Vida, Siemens Healthcare, Erlangen, Germany) after abstaining from caffeine and xanthine for 24 h. The CMR protocol is presented in Fig. 4 and typical sequence parameters are listed in Table 3. After the CMR examination, patients will be asked about symptoms experienced during breathing maneuvers and adenosine administration. They will also be asked about their preference for the stress testing method (adenosine or breathing maneuvers) during the CMR examination.

**Table 3:** Typical sequence parameters used in the scanning protocol of ADVOCATE-CMR study.

Type of CMR imaging	Planes	Sequence	Typical parameters
Cine	• LAX ○ 2CH ○ 3CH ○ 4CH • SAX stack	bSSFP	• In-plane resolution 1.5 × 1.5 mm^2^ • Slice thickness 5 mm • TE 1.6 ms • TR 3.7 ms • Matrix size 240 × 240* • FoV 320-400 mm* • FA 80° • GRAPPA/T-pat factor 2
Oxygenation-sensitive sequence	• SAX ○ Basal ○ Mid-ventricular	bSSFP	• In-plane resolution 1.9 × 2.3 mm^2^ • Slice thickness 10 mm • TE 1.73 ms • TR 3.45 ms • Matrix size 192 × 120* • FoV 320-400 mm* • FA 35° • GRAPPA/T-pat factor 2
T1 mapping	• SAX ○ Basal ○ Mid-ventricular ○ Apical	MOLLI 5(3)3	• In-plane resolution 1.4 × 2.1 mm^2^ • Slice thickness 8 mm • TE 1.06 ms • TR 2.54 ms • TI: Start T1 100 ms, increment 80 ms • Matrix size 256 × 169* • FoV 320-400 mm* • FA 35° • GRAPPA/T-pat factor 2
Quantitative perfusion	• SAX ○ Basal ○ Mid-ventricular ○ Apical	Saturation recovery turbo spoiled gradient echo	• In-plane resolution 2.3 × 2.3 mm^2^ • Slice thickness 8 mm • TE 1.18 ms • TR 2.64 ms • TS 100 ms • Matrix size 160 × 160 • FA 12° • GRAPPA/T-pat factor 2
Late gadolinium enhancement	• LAX ○ 2CH ○ 3CH ○ 4CH • SAX stack	2D segmented inversion recovery gradient echo	• In-plane resolution 1.8 × 2.4 mm^2^ • Slice thickness 8 mm • TE 1.2 ms • TR 3.1 ms • TI adjusted to null the myocardium (based on the modified look-locker approach) • Matrix size 192 × 144* • FoV 320-400 mm* • FA 10°

*2CH* two-chamber view, *2D* two-dimensional, *3CH* three-chamber view, *4CH* four-chamber view, *bSSFP* balanced steady-state free precession, *FA* flip angle, *FoV* field of view, *GRAPPA* generalized autocalibrating partially parallel acquisition, *LAX* long axis, *MOLLI* modified look-locker inversion recovery, *mSSFP* modified oxygenation-sensitive balanced steady-state free precession, *SAX* short axis, *TE* time of echo, *TI* time of inversion, *TR* time of repetition, *TS* time of saturation

* Depending on the patient’s size, increase phase FoV

#### 2.4.1 Pre-scan procedure (outside the scanner)

Every participant scheduled for a CMR scan will be checked to pass the routine MRI safety screening of Amsterdam UMC, which includes questions on past medical and surgical history and the presence of any implant, device, or metallic foreign body inside or at the body surface of the participant. Any patient who does not meet the safety requirements will be excluded from the study. Eligible study candidates will be instructed and practice the breathing maneuver, including 60 s of paced hyperventilation followed by a maximal voluntary breath-hold (for as long as possible, preferably 60 s). An instructional video is used to facilitate the instructions for patients.

#### 2.4.2 CMR acquisition of cine images

At the beginning of the CMR examination, localizer images will be obtained to define long-axis (LAX) and short-axis (SAX) planes. Cine images (LAX two-, three-, and four-chamber views and SAX) will be acquired with a balanced steady-state free precession (bSSFP) pulse sequence during breath-hold at end-expiration. Subsets of SAX images at the basal, mid-ventricular, and apical levels will be selected and used to ensure consistent slice positioning across all sequences used. SAX imaging planes will be determined using the end-systolic phase of LAX four-chamber cine images. Care will be taken to avoid inclusion of the left ventricular outflow tract in the basal slice.

#### 2.4.3 Acquisition of OS-CMR images

Baseline OS acquisition will be acquired using a modified OS bSSFP (mSSFP; [65]). This acquisition will take ∼10 s and will be performed in two SAX slices (basal and mid-ventricular) at end-expiration. The obtained images will be checked for slice location and the absence of significant artifacts. If necessary, baseline OS sequences will be repeated to achieve adequate image quality.

The following continuous acquisition will be performed using the same slice locations and sequence parameters. It will take 2 min, including 1 min of hyperventilation at a frequency of 30 breaths/min and 1 min of breath-hold at comfortable end-expiration. Patients will be guided through the breathing maneuvers by pre-recorded instructions played through the MRI audio system. After breathing maneuvers, patients will be allowed to breathe normally for 2–3 min without any image acquisition.

#### 2.4.4 CMR acquisition of T1 mapping

Native T1 mapping will be obtained at three image SAX planes (basal, mid-ventricular, and apical) using a modified look-locker inversion recovery (MOLLI) 5(3)3 sequence at rest (baseline) and after at least 3 min of constant intravenous infusion of adenosine at a dose of 140 μg/kg/min. To evaluate adenosine effectivity, heart rate increase and drop in blood pressure will be analyzed. With suspicion of insufficient effect of adenosine (heart rate increase less than 10 beats per minute), the dose will be increased first to 180 μg/kg/min for 2–3 min and, if ineffective, to 210 μg/kg/min for 2–3 min [66]. At the beginning of image acquisition, patients will be asked to hold their breath as long as possible and breathe slowly thereafter.

#### 2.4.5 CMR acquisition of first-pass perfusion and late gadolinium enhancement images

Myocardial first-pass perfusion images will be acquired using a saturation recovery turbo spoiled gradient-echo sequence at every heartbeat over 70 beats in three image SAX planes (basal, mid-ventricular, and apical). At start, two proton density-weighted images will be acquired at all three levels for correcting surface-coil SI inhomogeneity. In patients with a heart rate exceeding 80 beats per minute, the three slices will be acquired in 2 heartbeats/concatenations. Stress arterial input function (AIF) and perfusion images will be acquired after at least 3 min of constant intravenous infusion of adenosine at a dose of 140 μg/kg/min (or higher if necessary, as described above). AIF images will be acquired during intravenous administration of a pre-bolus of GBCA (DOTAREM®, Guerbet, Villepinte, France; 0.5 mmol/mL) at a dose of 0.0075 mmol/kg. Perfusion images will be acquired using a main bolus of GBCA (Dotarem, Guerbet, Villepinte, France; 0.5 mmol/mL) at a dose of 0.075 mmol/kg [31]. Patients will be asked to hold their breath as long as possible and breathe slowly thereafter. Rest AIF and perfusion images will be acquired using identical scanning parameters, pre-bolus and bolus doses, and slice location at least 10 min after adenosine administration.

Late gadolinium enhancement (LGE) images will be acquired 12–15 min after rest perfusion using a two-dimensional segmented inversion recovery gradient-echo pulse sequence.

#### 2.4.6 CMR image post-processing

All CMR images will be anonymized before the analysis. CMR post-processing and analysis will be conducted by readers who are blinded to the patients’ clinical data, including ICA results, and other CMR-derived results. Images will be analyzed using certified software equipped with a pixel-wise QP module and a prototype of dedicated software for the analysis of dynamic changes in myocardial oxygenation in OS-CMR images.

The QP module automatically corrects in-plane respiratory motion of the heart and surface coil-induced signal inhomogeneities (using proton density images), as described previously [67], [68]. Next, the system detects the AIF and myocardial ROIs to derive time-SI curves and identify key time points during first-pass contrast enhancement [69]. The software deconvolves the AIF and myocardial time-SI curves on a pixel-by-pixel basis to generate MBF estimates and obtain fully quantitative MBF maps [25]. Myocardial segmentation is done automatically [70]. Inappropriate automatic delineation of myocardial ROIs will be corrected by the operator. Quantitative analysis will include measurement of stress and rest MBF (mL/g/min), myocardial perfusion reserve (MPR; calculated as a ratio of MBF at stress over rest), relative MBF stress and rest (rMBF; calculated as a ratio of MBF to MBF of a remote segment showing the second highest value), and relative MPR (rMPR; calculated as a ratio of MPR to MPR of a remote segment showing the second highest value). These parameters will be calculated for all automatically determined myocardial segments according to the American Heart Association (AHA) 17-segment model, excluding apex [71]. In addition, the automated 32-segment model based on the 17-segment AHA model, excluding the apex and with epi- and endocardial subdivisions, will be applied.

Qualitative assessment of stress first-pass perfusion CMR and LGE images will be done by two independent level 3 CMR-certified experts under the supervision of a third level 3 expert with the highest experience in stress-perfusion CMR imaging. Scan quality will be graded as uninterpretable, poor, average, or good. First-pass stress-perfusion and LGE images will be visually analyzed according to the AHA 17-segment model, excluding the apex. Each myocardial segment will be assessed for the amount of stress-perfusion defect and LGE and scored according to a 5-point score: 0: 0%, 1: 1–25%, 2: 26–50%; 3: 51–75%; and 4: >75%. Presence and extent of ischemia and LGE will be scored by two (primary and secondary) independent level 3 CMR-certified experts. In case of discordance between the readers, the primary and secondary graders will evaluate the scan together. If discordance cannot be resolved, a third and final expert reader will evaluate the scan, and this assessment will be used for the analyses.

T1 maps will undergo quality control before post-processing, using the raw MOLLI images. Endocardial and epicardial contours will be placed manually, and partial volume effects from the blood and extra-myocardial tissues will be carefully avoided. Regional rest and stress T1 values will be measured according to the AHA 17-segment model, excluding apex. ∆T1 will be calculated as (stress T1 − rest T1)/rest T1 × 100%.

Before the post-processing of OS-CMR, the image quality will be assessed by a reader experienced in OS assessment. The OS analysis will be performed in end-systole to allow for the greatest contouring area. Epicardial and endocardial contours will be drawn by the software and (if necessary) corrected by the operator with careful attention to avoid inclusion of intracavitary blood, papillary muscles, or artifacts. B-MORE is calculated as a percent change in mean SI of the end-systolic images at 30 s (vasodilation) compared to 0 s of the breath-hold (vasoconstriction), according to the equation: ΔSI (%) = 100 × [(SI30s − SI0s)/SI0s] [65]. Other markers will be derived from an artificial intelligence analysis of the SI curves across the cardiac cycles during the post-hyperventilation breath-hold.

### 2.5 ICA with hemodynamic measurements

The time between ICA and CMR will be a maximum of 6 weeks. ICA is clinically indicated for the study participants. In addition to standard ICA, FFR, iFR, resting Pd/Pa, CFR, and IMR measurements of all main coronary arteries will be performed. All images and invasive measurements will be obtained and interpreted by experienced interventional cardiologists, blinded to the results of CMR. Details on the methodology of ICA and hemodynamic measurements are presented in Supplementary Material.

### 2.6 Follow-up CMR

To assess the impact of revascularization on myocardial perfusion and oxygenation, a follow-up CMR scan will be performed 3 months after ICA using the same protocol as the baseline scan to quantify changes in MBF, MBV, and myocardial oxygenation following coronary revascularization. In case of revascularization performed separately more than 1 day following ICA, the follow-up CMR will be performed 3 months after the (last) revascularization procedure.

### 2.7 Clinical follow-up

Data on clinical follow-up will be collected at 3, 6 months, 1 and 3 years after ICA (or after the [last] revascularization procedure if performed separately). Data on medical history since study inclusion, including SAQ-7 score, current pharmacotherapy, acute coronary syndromes, elective or emergency revascularization, and hospital admissions for cardiovascular causes, will be collected. Moreover, information on cardiovascular and all-cause death will be collected. If available, upon permission, the data will be collected from the electronic patient records. If electronic patient record will not be available or deemed insufficient, the data will be collected via telephone.

### 2.8 Definition of outcomes/events

#### 2.8.1 Diagnosis of obstructive CAD and CMD

For the primary endpoint analysis, hemodynamically significant (obstructive) CAD will be diagnosed if the FFR is ≤0.80 or the diameter stenosis is ≥90% (and if FFR was not obtained due to safety reasons) in at least one major coronary artery. Additional analyses will be performed for the FFR cut-off values of ≤0.75 and ≤0.70. For the secondary endpoint analyses, obstructive CAD will be defined as 1) iFR <0.9 or if a diameter stenosis ≥90% in at least one major coronary artery; 2) Pd/Pa ≤0.91 or if a diameter stenosis ≥90% in at least one major coronary artery. CMD will be diagnosed if invasively measured CFR <2.0. Additional analyses will be performed for CMD defined as CFR <2.5 or IMR ≥25. Definitions of other clinical outcomes and events, including cardiovascular death, myocardial infarction, ischemia-driven revascularization, and stroke, are presented in Supplementary Material.

### 2.9 Statistical considerations

#### 2.9.1 Sample size and power calculation

Sample size and power calculation were performed according to the primary endpoint. Based on the literature, we assumed the prevalence of obstructive CAD, as defined by FFR, in the study population to be 45% [72]. A sample with 74 from the positive group and 90 from the negative group achieves an 80% power to detect a difference of 0.09 between the area under the receiver operating characteristics curve (AUC) under the null hypothesis of 0.81 and an AUC under the alternative hypothesis of 0.9, using a two-sided z-test at a significance level of 0.05. The data are continuous responses [30], [72]. The AUC is computed between false positive rates of 0 and 1. The ratio of the standard deviation of the responses in the negative group to the standard deviation of the responses in the positive group is 1. Moreover, a sample of 75 from the positive group and 92 from the negative group achieves 80% power to detect a difference of 0.09 between a diagnostic test with an AUC of 0.81 and another diagnostic test with an AUC of 0.9 using a two-sided z-test at a significance level of 0.05. The correlation between the two diagnostic tests is assumed to be 0.6 for the positive group and 0.6 for the negative group. The drop-out rate in our study has been assumed 10%, which is comparable to other prospective diagnostic studies on stress-perfusion CMR and our previous experience in performing diagnostic studies on non-invasive imaging techniques (including CMR) in the detection of obstructive CAD [73], [74], [75]. Therefore, incorporation of the 10% drop-out to the sample size described above (74 from the positive group and 90 from the negative group, in total 164) results in a total sample size of 182 patients (which should also be sufficient to detect a significant difference between two diagnostic tests as described above).

#### 2.9.2 Statistical analysis

The statistical analysis will be performed using SPSS software package (IBM SPSS Statistics 20.0, Chicago, Illinois) and MedCalc (MedCalc Software 12.7.8.0, Mariakerke, Belgium). For the primary objective of the study, to validate the QP CMR against FFR (similarly for ΔT1 mapping and OS-CMR), first, intraclass correlation coefficients will be calculated, and second, Bland-Altman plots will be constructed. This will be performed for all comparisons between two continuous variables. Moreover, using FFR ≤0.8 as a definition of a hemodynamically significant coronary artery obstruction, AUCs will be determined for prediction of CAD using quantitative measures of QP CMR, ΔT1, and B-MORE, and perfusion defect score for conventional visual assessment of first-pass perfusion stress CMR. AUCs will be compared using the DeLong method. Diagnostic performance measures (sensitivity, specificity, PPV, NPV, and accuracy) will be calculated for the entire cohort, including 95% confidence interval. To prevent overestimation of the sensitivity, specificity, PPV, NPV, and accuracy, a balanced five-fold cross-validation will be performed. Comparison of sensitivity, specificity, and accuracy between different imaging modalities will be performed using McNemar’s test. Results will be considered statistically significant if p-value <0.05.

### 2.10 Data collection and monitoring

All data collected in the study will be recorded using a dedicated electronic case report form (eCRF). On-site monitoring will be performed by the local Clinical Monitoring Center (CMC, Amsterdam UMC). At the beginning of the study, a full monitoring schedule will be created, and an independent monitor will verify the completeness and quality of eCRF records by comparison with the source data. The number of any missing or uninterpretable diagnostic tests will be recorded. Adverse events are defined as any undesirable experience occurring to a subject during the study, whether or not considered related to study procedures. All adverse events reported spontaneously by the subject or observed by the investigator or the staff will be recorded. The investigator will report all serious adverse events (SAEs) to the accredited medical ethics committee that approved the protocol, except for SAEs that are unrelated to the CMR and/or ICA with FFR, as interpreted by the principle investigator. Due to the minor risk of an adverse event, an independent data safety monitoring board will not be appointed. Reporting of SAEs to the medical ethics committee is thought to be sufficient to monitor the safety of participating subjects.

## 3 Conclusions and implications

The ADVOCATE-CMR study is a single-center, observational, prospective, cross-sectional cohort study to validate QP CMR, ∆T1, and OS-CMR imaging against invasive gold standards (FFR, iFR, or resting Pd/Pa) for the detection of obstructive CAD. This is the first study comprehensively assessing and comparing head-to-head the diagnostic performance of a range of contrast- and non-contrast-based CMR imaging methods for the detection of FFR-defined obstructive CAD. Based on our results, we expect to establish a validated, time-efficient, and cost-effective diagnostic workflow available to a wide range of general CMR services performing CMR. Finally, these improvements may enable CMR to become an effective, potentially needle-free, non-invasive gatekeeper for ICA in patients with suspected obstructive CAD and may become an alternative for conventional invasive, radiation-based strategies.

## Study status

Currently including patients.

## Funding

This is an investigator-driven study.

## Author contributions

**Sonia Borodzicz-Jażdżyk:** Writing – original draft, Supervision, Resources, Project administration, Methodology, Formal analysis, Data curation, Conceptualization. **Geoffrey W. de Mooij:** Writing – review & editing, Project administration, Methodology, Data curation. **Mark B.M. Hofman:** Writing – review & editing, Resources, Methodology. **Alexander W. den Hartog:** Writing – review & editing, Methodology, Data curation. **Marco J.W. Götte:** Writing – review & editing, Supervision, Resources, Methodology, Conceptualization.

## Ethics approval and consent

The study is conducted in accordance with GCP, Medical Research Involving Human Subjects Act (WMO), and the Declaration of Helsinki (7th revision, October 2013). The study protocol has been approved by the local ethics committee (Medisch Ethische Toetsingscommissie Amsterdam UMC).

## Declaration of competing interests

The authors declare that they have no known competing financial interests or personal relationships that could have appeared to influence the work reported in this paper.
