# Supplementary material for: Advanced cardiac magnetic resonance imaging for assessment of obstructive coronary artery disease—ADVOCATE-CMR study rationale and design
Source: J Cardiovasc Magn Reson. 2025 Apr 25;27(2):101900. doi: 10.1016/j.jocmr.2025.101900 (PMC12175715; doi:10.1016/j.jocmr.2025.101900)
Supplement: Supplementary file 1 — Supplementary material [file mmc1.docx]

**Supplementary material**

### **ICA with hemodynamic measurements**

ICA will be performed via a trans-femoral or trans-radial approach according to the standard procedure with a biplane or monoplane cardiovascular X-ray system (Allura Xper FD 10/10, Philips Healthcare, Best, The Netherlands) in at least two orthogonal directions per evaluated coronary artery segment. Prior to contrast injection, 0.2 mL (200 µg) of intracoronary nitroglycerine will be administered to induce epicardial coronary vasodilation. All coronary physiological measurements will be obtained after diagnostic ICA. The 0.014-inch pressure-temperature sensor guidewire will be zeroed and equalized to aortic pressure, and then the pressure sensor will be positioned at the distal segment of a target vessel. Resting Pd/Pa will be measured as a ratio between proximal (Pa) and distal coronary pressures (Pd) over entire resting cycle period. Three injections of 3 mL of room-temperature saline down the coronary artery will be done to assess resting mean transit time (T_mn_). To induce maximal coronary hyperemia, intracoronary (150 µg) or intravenous (140 µg/kg/min) adenosine infusion will be used. During sustained hyperemia, distal coronary artery pressure and aortic pressure will be obtained and FFR will be calculated as the ratio of the mean distal intracoronary pressure, to the mean arterial pressure measured by the coronary catheter. FFR will be obtained in all major coronary arteries, except for occluded or subtotal lesions ≥90% diameter stenosis. Additionally, three injections of 3 mL of room-temperature saline will be made to measure the hyperemic T_mn_. CFR will be calculated as resting T_mn_ divided by hyperemic T_mn_. IMR will be calculated as the distal coronary pressure at maximal hyperemia divided by the inverse of the hyperemic T_mn_. After measurements, the pressure wire will be pulled back to the guide catheter, and the presence of pressure drift will be assessed. iFR will be calculated as the mean pressure distal to the stenosis divided by the mean aortic pressure during the diastolic wave-free period. Clinical decision making and subsequent coronary stenting will be based on ICA findings and physiologic measurements (FFR, iFR), which is the golden standard and is left at the discretion of the interventional cardiologist.

**Definition of outcomes/events**

***Cardiovascular death***

Cardiovascular (CV) death includes death resulting from an acute myocardial infarction, sudden cardiac death, death due to heart failure, death due to stroke, death due to CV procedures, death due to CV hemorrhage, and death due to other CV causes (according to the 2017 Cardiovascular and Stroke Endpoint Definitions for Clinical Trials, developed by the Standardized Data Collection for Cardiovascular Trials Initiative and the US Food and Drug Administration (FDA) [1].

***Myocardial infarction***

Myocardial infarction will be diagnosed according ESC/ACCF/AHA/WHF 4^th^ Universal Definition of Myocardial Infarction, in general when there is evidence of myocardial necrosis in a clinical setting consistent with myocardial ischemia [1, 2].

***Ischemia-driven revascularization***

Ischemia-driven coronary revascularization endpoint will include all coronary revascularization that were performed in the context of myocardial infarction and those for worsening symptoms in combination with evidence of myocardial ischemia [3]. The definition of the latter will require: 1) a history of new or worsening symptoms consistent with myocardial ischemia that is not provoked by factors such as anemia, tachyarrhythmia, cold, unusual effort, sepsis or non-compliance with anti-anginal medication; 2) no documented rise in serum troponin suggestive of myocardial infarction; 3) angiographic evidence of a culprit lesion; 4) revascularization.

***Stroke***

Stroke will be defined as an acute episode of focal or global neurological dysfunction caused by brain, spinal cord, or retinal vascular injury as a result of hemorrhage or infarction (according to the 2017 Cardiovascular and Stroke Endpoint Definitions for Clinical Trials, developed by the Standardized Data Collection for Cardiovascular Trials Initiative and the US Food and Drug Administration (FDA) [1].

**References**

1. Hicks, K.A., et al., *2017 Cardiovascular and Stroke Endpoint Definitions for Clinical Trials.* J Am Coll Cardiol, 2018. **71**(9): p. 1021-1034.

2. Thygesen, K., et al., *Fourth Universal Definition of Myocardial Infarction (2018).* J Am Coll Cardiol, 2018. **72**(18): p. 2231-2264.

3. Nidorf, S.M., et al., *Colchicine in Patients with Chronic Coronary Disease.* N Engl J Med, 2020. **383**(19): p. 1838-1847.
